# Multiple pathways through which the gut microbiota regulates neuronal mitochondria constitute another possible direction for depression

**DOI:** 10.3389/fmicb.2025.1578155

**Published:** 2025-04-17

**Authors:** Hongyi Zhao, Xiongfeng Qiu, Shuyu Wang, Yi Wang, Li Xie, Xiuwen Xia, Weihong Li

**Affiliations:** ^1^School of Basic Medical Science, Chengdu University of Traditional Chinese Medicine, Chengdu, China; ^2^School of Health Preservation and Rehabilitation, Chengdu University of Traditional Chinese Medicine, Chengdu, China; ^3^Sichuan College of Traditional Chinese Medicine, Mianyang, China

**Keywords:** gut microbiota, depression, neuronal mitochondria, immune, intestinal-brain axis

## Abstract

As a significant mental health disorder worldwide, the treatment of depression has long faced the challenges of a low treatment rate, significant drug side effects and a high relapse rate. Recent studies have revealed that the gut microbiota and neuronal mitochondrial dysfunction play central roles in the pathogenesis of depression: the gut microbiota influences the course of depression through multiple pathways, including immune regulation, HPA axis modulation and neurotransmitter metabolism. Mitochondrial function serves as a key hub that mediates mood disorders through mechanisms such as defective energy metabolism, impaired neuroplasticity and amplified neuroinflammation. Notably, a bidirectional regulatory network exists between the gut microbiota and mitochondria: the flora metabolite butyrate enhances mitochondrial biosynthesis through activation of the AMPK–PGC1α pathway, whereas reactive oxygen species produced by mitochondria counteract the flora composition by altering the intestinal epithelial microenvironment. In this study, we systematically revealed the potential pathways by which the gut microbiota improves neuronal mitochondrial function by regulating neurotransmitter synthesis, mitochondrial autophagy, and oxidative stress homeostasis and proposed the integration of probiotic supplementation, dietary fiber intervention, and fecal microbial transplantation to remodel the flora–mitochondrial axis, which provides a theoretical basis for the development of novel antidepressant therapies targeting gut–brain interactions.

## 1 Introduction

Depression is a disorder that severely affects the mental health of the global population and is characterized by persistent low mood, loss of interest and cognitive dysfunction (GBD 2017 Disease and Injury Incidence and Prevalence Collaborators, 2018; COVID-19 Mental Disorders Collaborators, 2021; Salari et al., 2020). Globally, depression is one of the leading causes of mental disability. According to the World Health Organization (WHO), the global prevalence of depression is approximately 4.4%, which means that more than 300 million people worldwide suffer from depression (Xu et al., 2024). In addition, depression is one of the major causes of suicide deaths, with nearly 800,000 people worldwide dying by suicide each year (World Health Organization, 2021). The current treatment of depression faces three core challenges: first, a severe undertreatment rate due to stigma, disease cognitive bias, and insufficient healthcare resources; second, limitations of existing therapies, including erratic drug efficacy, significant side effects, and low accessibility to psychotherapies; and third, a high rate of recurrence, which exacerbates the risk of chronicity due to poor adherence and difficulties in the management of residual symptoms (Lassen et al., 2024; Kajumba et al., 2024; Alang and McAlpine, 2020). Its pathogenesis is more complex, with molecular mechanisms involving multiple factors, such as neurotransmitter imbalance, decreased neuroplasticity and the inflammatory response (Peng et al., 2015). In recent years, an increasing number of studies have shown that the gut microbiota can influence the development of depression through immune and nervous system pathways, the HPA axis, and neurotransmitter pathways (Zhao et al., 2024). In particular, the gut microbiota regulating neuronal mitochondrial function has become a new research hotspot. As the energy factories of cells, mitochondria play crucial roles in neuronal survival, function and plasticity (Klemmensen et al., 2024). Studies have shown that neuronal mitochondrial function is often impaired in depressed patients, which may be closely related to gut microbiota dysbiosis (Kunugi, 2021). The gut microbiota directly or indirectly affects the health of neuronal mitochondria through multiple mechanisms, such as regulating immune responses and producing neurotransmitters and various metabolites, which in turn affects the occurrence and symptomatic manifestations of depression (Qiao et al., 2024). The aim of this study was to investigate the mechanisms by which the gut microbiota modulates neuronal mitochondrial function through multiple pathways, which in turn affects depression, and to elucidate the connections between the gut microbiota and brain function. Through these studies, we hope to provide new ideas and strategies for the prevention, early diagnosis, and individualized treatment of depression.

## 2 Gut microbiota and depression

The gut microbiota is closely related to human health, and the effects of the gut microbiota can extend to the brain through a variety of pathways. Stress and emotions can affect gut physiology and alter the microbiota through the release of stress hormones or sympathetic neurotransmitters. Conversely, neurotransmitters secreted by the gut microbiota can influence brain-related functions through humoral and neuromodulation (Collins et al., 2012; Young, 2017). Many studies at home and abroad have confirmed that the diversity of gut microbiota influences human metabolism, the gastrointestinal tract, and psychology and is closely related to neurological disorders such as depression, Parkinson’s disease, Alzheimer’s disease, and other neurological disorders (Flint et al., 2012; Cryan and O'Mahony, 2011; Lyte, 2014; De Palma et al., 2017; Bruce-Keller et al., 2015; O'Hara and Shanahan, 2006). The gut microbiota plays a very important role in the development of depression (Morais et al., 2021).

### 2.1 The gut microbiota influences the development of depression through multiple pathways

The gut microbiota affects the occurrence and development of depression by regulating the immune system, the HPA axis (hypothalamic–pituitary–adrenal axis), and the metabolism of neurotransmitters, revealing the key role of the gut-brain axis in the regulation of mood and behavior.

#### 2.1.1 Immune and nervous system pathways

Short-chain fatty acids (SCFAs), metabolites of the gut microbiota, alleviate neuroinflammation by enhancing intestinal barrier function, inhibiting the release of proinflammatory factors, and modulating microglial cell activity (Cryan and Dinan, 2012; Silva et al., 2020; Parada Venegas et al., 2019); the leaky gut effect triggered by dysbiosis allows endotoxins to enter the bloodstream, which triggers brain dysfunction via vagal nerve and cytokine infiltration (Feng et al., 2018; Macpherson et al., 2012; Alcocer-Gómez et al., 2014; Slyepchenko et al., 2015; Wong et al., 2016). Moreover, specific strains (e.g., *Bifidobacterium bifidum* and Lactobacillus) improve neuroplasticity by upregulating BDNF expression (Sarkar et al., 2016). Mucinophilic Ackermannia, whose metabolites enhance intestinal barrier function, reduce the release of inflammatory factors (IL-6, TNF-*α*), and ameliorate neuroinflammation by activating mitochondrial energy metabolism (Ghaffari et al., 2023). In addition, the flora affects neurotransmitter homeostasis by regulating the tryptophan-5-HT metabolic axis, and its metabolic imbalance may lead to the accumulation of neurotoxic products, forming a multilayered regulatory network from the gut to the brain and ultimately triggering abnormalities in depression-related neural circuits.

#### 2.1.2 HPA axis pathway

Stress (stress) exposure is an important factor that triggers or aggravates depression (He et al., 2024). The HPA (hypothalamic–pituitary–adrenal axis) axis has been shown to alter brain function and cognitive behavior by affecting the gut microbiota. Depressed patients often exhibit hyperactivation of the HPA axis, resulting in elevated cortisol levels. During stress, the cerebral cortex activates the HPA axis, increasing serum cortisol and CRH levels in the cerebrospinal fluid. CRH and its receptors in the colonic mucosa affect intestinal epithelial cells, impairing intestinal barrier function, which in turn affects the immune and nervous systems through the intestines, leading to abnormalities in brain function and cognitive behavior (Foster and McVey Neufeld, 2013). Treatment of rats separated from their mothers with probiotics (Lactobacillus spp.) during early stress was found to normalize basal CORT levels and prevent the associated hyperreactivity of the HPA axis (Luna and Foster, 2015).

#### 2.1.3 Neurotransmitter pathways

**Figure 1:**
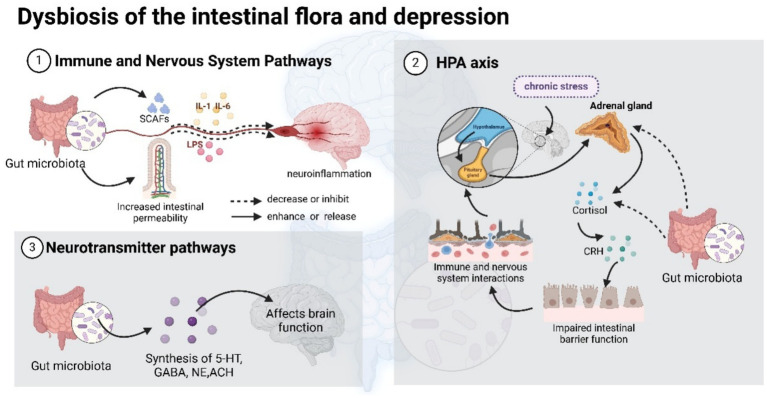
Multiple pathways through which the gut microbiota influences depression.

Recently, the neurotransmitter signaling interference hypothesis has been shown to exist between the gut microbiota and depression. Lactobacillus can secrete acetylcholine; Bacillus and *Serratia marcescens* can secrete dopamine (Averina et al., 2020). Synthetic pathways for tyrosine, epinephrine, and catecholamine cofactors have been reported in *E. coli* and other bacteria (Pellegrini et al., 2020). 5-HT, a neurotransmitter that is particularly critical in depression, is one of the key metabolites derived from tryptophan by the metabolism of the gut microbiota, and tryptophan is the only precursor for the synthesis of 5-HT (Xue et al., 2023). In turn, the main sites of 5-HT synthesis are enterochromaffin cells in the gut and the nucleus accumbens in the brainstem. 5-HT released into the synaptic gap is rapidly carried back to presynaptic neurons by the 5-hydroxytryptamine transporter protein (SERT) (Gao et al., 2020; Pourhamzeh et al., 2022). Other studies have shown that probiotics are also capable of producing a variety of neurotransmitters, such as GABA and NE (Dinan et al., 2013), and Prof. Jeroen Raes’ team investigated the correlation of microbiome profiles with host quality of life and depression, which revealed that gut–brain module analysis of fecal macrogenomes revealed a positive correlation between the microbial synthesis potential of the dopamine metabolite 3,4-dihydroxyphenylacetic acid and psychological quality of life and suggested a potential role for *γ*-aminobutyric acid produced by the gut microbiota in depression (Valles-Colomer et al., 2019). All of these transmitters are active compounds that potentially interact with the host and influence brain function (Figure 1).

In summary, the gut microbiota affects depression by modulating immune and nervous system pathways, stress response pathways (including hyperactivation of the HPA axis), and neurotransmitter pathways. These findings provide possible avenues for the development of new therapeutic strategies for depression, including probiotics, dietary modifications, and fecal microbial transplantation (Qiao et al., 2024).

### 2.2 Bidirectional mitochondria–microbiota regulation

Bidirectional mitochondria–microbiota regulation refers to mutually influencing and regulating relationships that exist between mitochondria and the gut microbiota. Mitochondrial dysfunction can alter the composition of the gut microbiota, and changes in the gut microbiota can in turn affect mitochondrial function (Wu et al., 2021).

On the one hand, mitochondrial dysfunction directly alters the intestinal microenvironment: abnormal mitochondrial energy metabolism can lead to hypoxia and oxidative stress in intestinal epithelial cells, contributing to the proliferation of pathogenic bacteria and inhibiting the colonization of commensal SCFA-producing bacteria, exacerbating the imbalance of the flora (Mafra et al., 2019). On the other hand, gut microbial metabolites modulate mitochondrial function through multiple pathways; for example, butyrate enhances mitochondrial biosynthesis and improves neuronal energy metabolism through activation of the AMPK-PGC1α pathway, whereas tryptophan derivatives protect neuronal cells from oxidative damage by scavenging mitochondrial ROS through activation of the Nrf2 pathway (Rekha et al., 2024; Dinkova-Kostova and Abramov, 2015). In addition, eosinophilic Ackermannia can regulate host mitochondrial autophagy and maintain cellular homeostasis by secreting outer membrane vesicles (Macchione et al., 2019). This bidirectional interaction suggests that targeting mitochondrial–microbial interactions may be a novel strategy for depression treatment.

## 3 Mitochondria and depression

In the brains of healthy individuals, mitochondria play important physiological roles by maintaining BBB permeability (Liu et al., 2024), maintaining hippocampal neuronal homeostasis and stabilizing glial cell function, in addition to functions such as adenosine triphosphate (ATP) generation, reactive oxygen species (ROS) production and ionic homeostasis maintenance (Yang et al., 2023). In contrast, reduced neurogenesis, impaired synaptic plasticity and abnormal neuronal network function are common in the hippocampus of patients with depression (MDD) (Allen et al., 2018). ATP consumption is high in the brain (Caruso et al., 2019), and mitochondria are the providers of ATP within the brain, which is found mainly in the dendrites and synaptic terminals of neurons (Du et al., 2008; Song et al., 2024). Decreased bioenergetic function and mitochondrial dysfunction during times of increased metabolic demand are important risk factors for psychiatric disorders (Manji et al., 2012; Gardner and Boles, 2011; Klinedinst and Regenold, 2015). Mitochondrial dysfunction leads to ATP deficiency, increased reactive oxygen species (ROS), and oxidative stress (Murphy and Hartley, 2018), which affects neurobiological processes, alters synapses, increases apoptosis, and may trigger mood disorders (Andreazza and Young, 2014; Caruso et al., 2019). Therefore, mitochondrial dysfunction is closely related to the development and treatment of mood disorders as well as disease progression and is an important target for potential treatment of mood disorders (Andreazza and Young, 2014; Kato, 2017; Bansal and Kuhad, 2016). Mitochondria play crucial roles in neurogenesis; they are not only the main source of neuronal energy but also regulate apoptosis, neuroinflammation, oxidative stress, free radical production, and calcium ion homeostasis (Johri and Beal, 2012). Mitochondrial dysfunction also includes the deletion of coenzymes and cofactors and changes in membrane potential, apoptosis, and inflammatory molecules, all of which disrupt mitochondrial structure and function, which in turn affects autophagic processes, leading to impaired interneuronal signaling and diminished synaptic growth and plasticity, all of which are key mechanisms in the pathogenesis of depression (Ben-Shachar and Laifenfeld, 2004).

### 3.1 Mitochondrial dysfunction affects neurogenesis

Mitochondria provide energy support for neurogenesis through oxidative phosphorylation, which is particularly critical in energy-intensive processes such as synapse formation, axon extension and synaptic transmission (Shabbir et al., 2021; Guo et al., 2021). Mitochondrial dysfunction (e.g., abnormal oxidative phosphorylation) directly leads to insufficient ATP production, interferes with the metabolic activities of neural progenitor cells, disrupts neurogenesis, and ultimately triggers dysfunctional mood regulation (Princz et al., 2018; Brunetti et al., 2021; Kausar et al., 2018). Mitochondrial division and fusion are necessary to maintain functional and morphological stability, and abnormal mitochondrial dynamics affect neuronal growth, synaptic plasticity, and network formation (Cagalinec et al., 2016). Elevated levels of Drp1 phosphorylation lead to excessive mitochondrial division, generating fragmented mitochondria, which weakens the synaptic terminal energy supply and calcium buffering, triggering aberrant presynaptic vesicle release and long-term potentiation (LTP) impairment (Grel et al., 2023); moreover, chronic stress downregulates Mfn2 expression to inhibit mitochondrial fusion, disrupting the functional synergism of the mitochondrial network and leading to reduced dendritic complexity and abnormal synaptic pruning, which are associated with hippocampal memory deficits and depressive-like behaviors (Filadi et al., 2018). Notably, mitochondrial transport in neurons is bidirectional. If the reverse transport mechanism is disturbed, it may block intersynaptic signaling and further weaken the adaptability of neural networks (Kim et al., 2022; Fukumitsu et al., 2016). Studies have shown that targeting kinetic homeostasis restores synaptic plasticity and improves depressive symptoms, suggesting its potential as a new strategy for treatment (Xu et al., 2023).

### 3.2 Mitochondrial dysfunction affects neuroapoptosis

Mitochondrial dysfunction has a significant effect on neuronal apoptosis (Lopriore et al., 2022). When mitochondrial damage is excessive and unrepairable, cells initiate apoptotic mechanisms that disrupt neural homeostasis and health (Wu et al., 2019). Inadequate energy supply, oxidative stress, and dysregulation of calcium homeostasis due to mitochondrial dysfunction further exacerbate neuronal damage (Song et al., 2021). Excessive reactive oxygen species (ROS) generated by oxidative stress can induce mitochondrial dysfunction, further exacerbating energy metabolism disorders and imbalances in intracellular redox homeostasis, which ultimately leads to neuronal apoptosis or necrosis and neuronal damage (Moris et al., 2017). Mitochondria play an important role in Ca^2+^ signaling. They not only regulate the intracellular Ca^2+^ concentration but also act as buffers and sensors involved in the regulation of neuronal excitability and cellular physiological functions. Mitochondria regulate energy production and neuronal excitability through Ca^2+^ uptake, and once mitochondrial function is impaired, excessive accumulation of Ca^2+^ disrupts Ca^2+^ homeostasis, ultimately leading to apoptosis (Pinto et al., 2015). In addition, mitochondrial dynamics are critical for neuronal development. Mitochondrial division and fusion are necessary to maintain functional and morphological stability, and abnormal mitochondrial dynamics affect neuronal growth, synaptic plasticity, and network formation.

In summary, mitochondrial dysfunction significantly affects neuron generation, development, and synaptic plasticity through mechanisms such as insufficient energy supply, oxidative stress, and dysregulation of calcium homeostasis, leading to neuronal damage and death. These mechanisms play important roles in the pathogenesis of depression and may lead to mood regulation and cognitive dysfunction.

### 3.3 Mitochondrial dysfunction affects neuroinflammation

Mitochondria are not only the energy factories of neurons but also involved in the regulation of immune responses. Through interactions with pattern recognition receptors (e.g., NLRP3 inflammatory vesicles), mitochondria can regulate neuroinflammatory responses and influence the immune environment of the nervous system (Zhong et al., 2016; Gong et al., 2020; Zhou et al., 2011). Damaged mitochondria are also capable of releasing multiple molecular patterns that activate inflammatory responses and trigger neuronal cell damage (Casaril et al., 2021). Activated microglia transmit inflammatory signals to astrocytes and neurons by releasing fragmented mitochondria, which may lead to impaired ATP synthesis within neurons and reduced inner mitochondrial membrane potential, thereby affecting normal neuronal function (Joshi et al., 2019). Mitochondria play a key role in maintaining intracellular calcium homeostasis (Bravo-Sagua et al., 2017). In the presence of mitochondrial dysfunction, intracellular calcium ion levels may increase, which contributes to the activation of microglia and astrocytes, further exacerbating neuroinflammation (Singh, 2022). Mitochondrial DNA (mtDNA) is an important signaling molecule in the inflammatory response (West and Shadel, 2017). When mitochondrial function is impaired or ruptured, mtDNA can be released into the extracellular space or cytoplasm. These exogenous mitochondrial DNAs can activate immune responses and trigger neuroinflammation through pathways such as the Toll-like receptor (TLR9) (Oka et al., 2012). In addition, mitochondrial DNA can induce inflammatory cytokines through the cGAS–STING signaling pathway, further exacerbating symptoms of depression (Qing et al., 2020). In summary, mitochondria further exacerbate neuroinflammation by interacting with pattern recognition receptors, increasing Ca^2+^ concentrations, releasing mitochondrial DNA (mtDNA), and inducing inflammatory factors to activate the immune response, which in turn affects neuronal function and promotes the activation of microglia and astrocytes.

**Figure 2:**
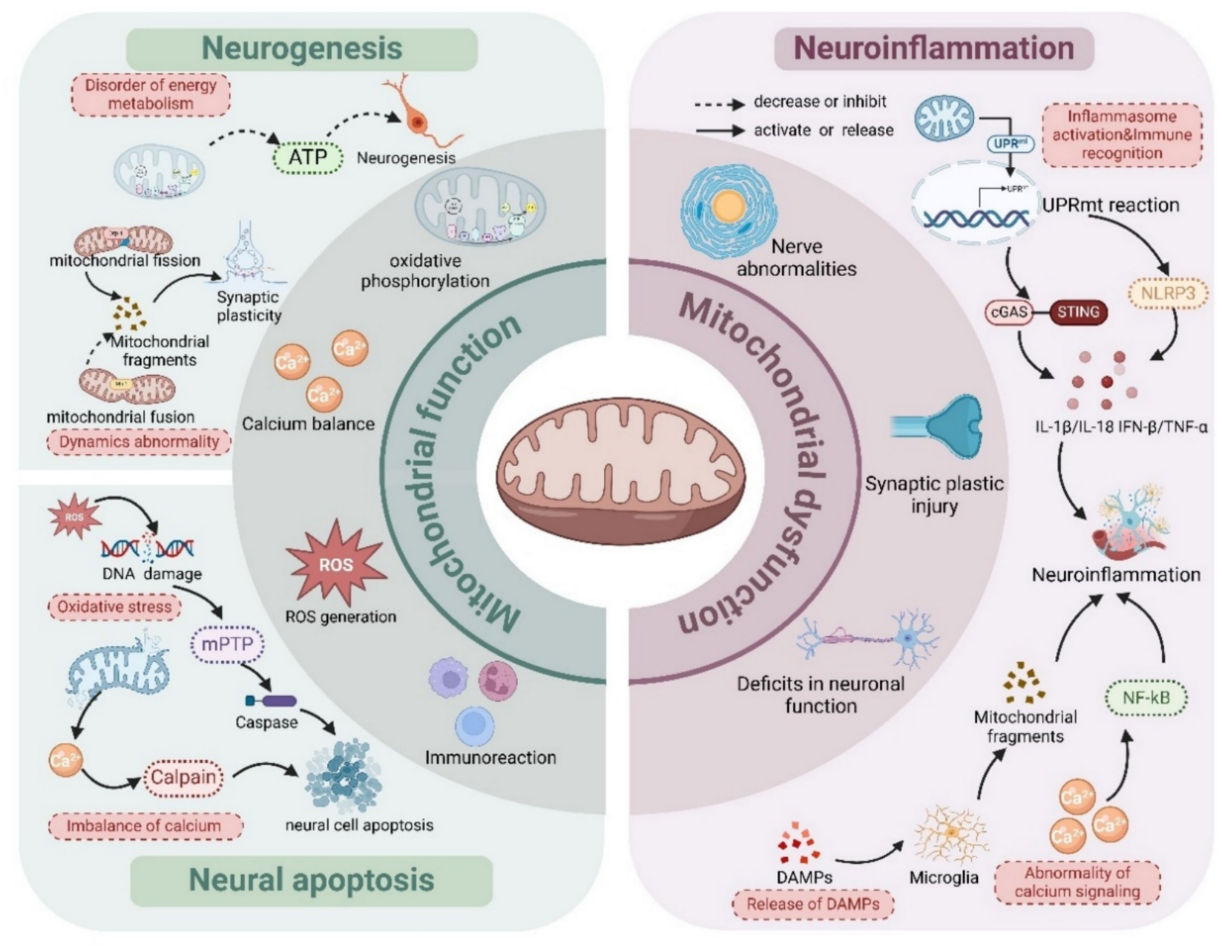
Mitochondrial dysfunction affects nerves.

Taken together, mitochondria significantly influence the course of neuroinflammation by interacting with pattern recognition receptors, increasing Ca^2+^ concentrations, releasing mitochondrial DNA, and activating immune responses, which promote the activation of microglia and astrocytes, further exacerbating neuroinflammatory responses. These studies reveal the important role of mitochondrial dysfunction in the pathogenesis of depression, particularly in the modulation of neuroinflammation and impairment of neuronal function (Figure 2).

### 3.4 Epigenetic regulation of mitochondrial genes

Epigenetic regulation of mitochondrial genes involves a variety of mechanisms, including DNA methylation, histone modification and the role of noncoding RNAs. Methylation levels of key mitochondrial DNA genes were found to be significantly elevated in the peripheral blood and brain tissues of depressed patients, which may exacerbate oxidative phosphorylation dysfunction and energy metabolism defects by inhibiting the transcription of NADH dehydrogenase and cytochrome C oxidase (FC Lopes, 2020). In addition, epigenetic modifications of nuclear-encoded mitochondria-related genes are also involved in regulation, as demonstrated by studies confirming that promoter hypermethylation of PGC-1α, a master regulator of mitochondrial biosynthesis, leads to downregulation of its expression, reduces mitochondrial production, and correlates with abnormal metabolism in the prefrontal cortex in depressed patients (Halling and Pilegaard, 2020) and that HDAC inhibitors activate SIRT3 by increasing the level of acetylation of histone H3K9, enhancing the antioxidant capacity and improving depressive-like behavior (He et al., 2023). These epigenetic changes may serve as a molecular bridge between environmental stress and mitochondrial dysfunction, providing new directions for targeting DNA demethylation or histone modification to treat depression (Yuan et al., 2023). 2.5 Antidepressant therapy targeting mitochondrial dysfunction.

Studies have shown that improving mitochondrial function can alleviate depressive symptoms through multiple pathways: mitochondrial transplantation alleviates neuroinflammation by increasing BDNF expression, increasing ATP synthesis, and decreasing oxidative stress (Wang et al., 2019). Ketamine, an antagonist of the N-methyl-D-aspartate (NMDA) receptor, has been shown to have antidepressant properties, and its rapid treatment may be achieved by decreasing ROS production and increasing the expression of OXPHOS-related enzymes (Corriger and Pickering, 2019; Kawazoe et al., 2022). By increasing the number of autophagic vesicles and increasing the expression of mitochondrial endosomal membrane uncoupling protein 2 (UCP2), fluoxetine promotes mitochondrial autophagy, with reduced ROS production and the upregulation of mitochondrial biogenesis-related genes to alleviate depression (Zeb et al., 2022). Other drugs, such as lamotrigine, have also been shown to inhibit the toxic effects of rotenone (a cytotoxic agent that inhibits mitochondrial electron transport chain complex I), prevent the opening of the mitochondrial permeability transition pore, increase glutathione levels, and maintain the mitochondrial membrane potential (Giménez-Palomo et al., 2021). In addition, repetitive transcranial magnetic stimulation significantly reduces synaptic loss and neuronal degeneration and inhibits the mitochondrial apoptotic pathway, effectively preserving the integrity of the mitochondrial membrane (Vucic et al., 2013). In summary, improving central mitochondrial energy metabolism disorders can effectively alleviate the mental and physical symptoms of depression and antioxidative stress, and scavenging ROS can reduce neuronal damage and apoptosis, protect neurons, and treat depression.

## 4 Multiple pathways regulated by the gut microbiota in neuronal mitochondria affect depression

In recent years, researchers have shown that the gut microbiota may regulate neuronal mitochondrial function to intervene in depression (Loh et al., 2024; Ribeiro et al., 2020; Yao et al., 2023), a finding that provides a new perspective on the prevention and treatment of depression.

### 4.1 There is a close association between the gut microbiota and mitochondria

In modern biology, bacteria and mitochondria may share the same phylogenetic history, and according to endosymbiotic theory, human mitochondria are descendants of microorganisms, and primitive mitochondria are believed to be ancient bacterial endosymbionts from which all the mitochondria of eukaryotic cells originated (Bajpai et al., 2018). The ability of host mitochondria to influence the diversity of the gut microbiota through the release of reactive oxygen species (ROS) suggests that the gut microbiota and mitochondria are indeed capable of generating biological “crosstalk” to influence health and disease (Yardeni et al., 2019). A recent study revealed that mitochondrial genotypes are associated with the composition of the mouse gut microbiota and that mitochondria, a genetically functional chimera thought to be the ancestor of Methanobacterium, are sensitive to the antibiotic chloramphenicol as an operant inhibitor, suggesting that the gut microbiota and mitochondria exhibit adaptive interactions (Dantzer et al., 2008). A team of researchers demonstrated the mechanism of interaction between flora and mitochondria through reactomics, screened a total of 2,626 *in vivo* metabolites, and found that 325 out of 437 metabolites from mitochondria overlapped with the metabolites of the gut microbiota (Thiele et al., 2013).

### 4.2 Multiple pathways regulation of neuronal mitochondria by the gut microbiota

#### 4.2.1 Metabolite pathways

The gut microbiota metabolize SCFAs (Koh et al., 2016) (e.g., butyric acid, acetic acid, and propionic acid) through fermentation of dietary fibers and undigested carbohydrates, and SCFAs can enter the brain through the blood–brain barrier. Butyric acid, the most important short-chain fatty acid, is an important energy substrate for neuronal mitochondria, which increases the efficiency of oxidative phosphorylation, regulates mitochondrial autophagy (mitophagy), and scavenges damaged mitochondria (Tang et al., 2011), which in turn increases the energy supply of neurons. Sodium butyrate is able to regulate intracellular signaling pathways by binding to G protein-coupled receptors, thereby affecting mitochondrial function and autophagy (Zhou et al., 2021; Caetano et al., 2023). Table 1 summarizes the major derived metabolites of the microbiota and their mitochondrial targets.

**Table 1:** Microbiota-derived metabolites and their mitochondrial targets.

Metabolite	Mitochondrial action
Butyrate	Inhibition of histone deacetylase (HDAC) enhances mitochondrial bioenergetics and oxidative phosphorylation (OXPHOS) (Simão et al., 2024); modulation of mitochondrial metabolism in immune cells via GPR109A receptor (Nikolova et al., 2021).
Kynurenine	The metabolite quinolinic acid (QUIN) induces mitochondrial ROS accumulation and impairs mitochondrial membrane potential through activation of NMDA receptors; kynurenic acid (KYNA) antagonizes glutamate receptors and reduces oxygen chemical stress (Fries et al., 2023).
Secondary bile acids (DCA/LCA)	Interferes with mitochondrial membrane stability, induces ROS generation and mtDNA damage; inhibits mitochondrial complex I activity and reduces ATP synthesis (Simão et al., 2024).
Tryptophan derivatives (5-HT precursors)	Tryptophan is metabolized by microorganisms to produce 5-hydroxytryptamine (5-HT), which regulates neurotransmitter release via mitochondrial calcium signaling; 5-HT deficiency is associated with impaired mitochondrial energy metabolism (Hoban et al., 2017).
Short-chain fatty acids (SCFAs)	Participates in the TCA cycle as a mitochondrial substrate to generate ATP; propionate inhibits HDAC via GPR41 and promotes PGC-1α-mediated mitochondrial biogenesis (Nikolova et al., 2021).
Gamma-aminobutyric acid (GABA)	Regulation of neuronal excitability via mitochondrial glutamate-GABA shuttle; inhibition of mitochondrial ROS production and maintenance of synaptic stability (Fries et al., 2023).
Lipopolysaccharide (LPS)	Activation of TLR4 signaling induces mitochondrial ROS release and mtDNA leakage and activates NLRP3 inflammatory vesicles (Simão et al., 2024).

#### 4.2.2 Neurotransmitter pathways

The gut microbiota activates the vagus nerve through the production of neuroactive substances such as *γ*-aminobutyric acid (GABA) and 5-hydroxytryptamine (5-HT), which affect the activity of brain neurons and their mitochondrial function (Cryan and Dinan, 2012). Bifidobacteria and Lactobacillus can directly synthesize GABA and norepinephrine, and GABA crosses the mitochondrial membrane and regulates the citric acid cycle (Zhu et al., 2022). In addition, GABA regulates the calcium ion concentration in the mitochondria, which in turn affects oxidative phosphorylation and energy production (Sittipo et al., 2022). In addition, norepinephrine is involved in the stress response and increases energy metabolism, and it also crosses the blood–brain barrier into the brain, where it regulates the redox state within neuronal mitochondria (O'Donnell et al., 2012). 5-HT produced by the gut microbiota can affect the central nervous system via the vagus nerve, further regulating neuronal activity and the mitochondrial membrane potential (Wang et al., 2023). Approximately 95% of 5-HT is produced by the gut microbiota (Terry and Margolis, 2017), and 5-HT is an upstream regulator of mitochondrial biogenesis and function in cortical neurons (Fanibunda et al., 2019). In addition, 5-HT receptor activation can regulate mitochondrial endosomal function and increase the efficiency of oxidative phosphorylation via the cAMP pathway (Chen et al., 2007). The gut microbiota can generate dopamine by metabolizing tyrosine (Strandwitz, 2018). High dopamine concentrations induce mitochondrial dysfunction in the brain through reduced mitochondrial respiratory control and loss of membrane potential (Czerniczyniec et al., 2010).

#### 4.2.3 Immune pathways

**Figure 3:**
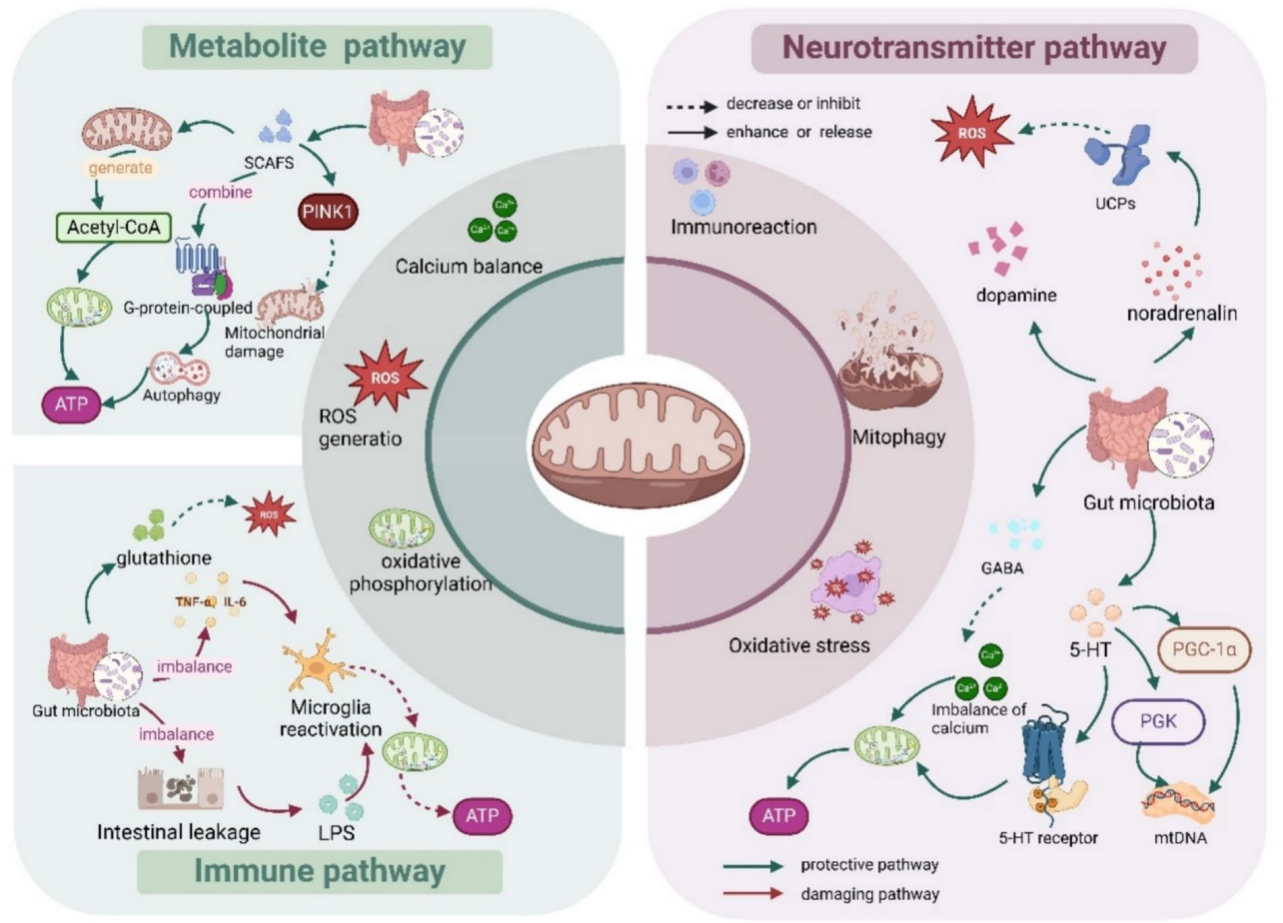
The gut microbiota regulates neuronal mitochondria via multiple pathways.

An imbalance of the gut microbiota (e.g., dysbiosis) may lead to increased intestinal permeability (i.e., leaky gut phenomenon), allowing endotoxins (e.g., lipopolysaccharides, LPS) to enter the brain through the blood circulation and activate microglia and astrocytes, leading to neuroinflammation (Di Vincenzo et al., 2024), which disrupts neuronal mitochondrial function, leading to oxidative stress and energy metabolism disorders (Harland et al., 2020). Moreover, chronic inflammation increases the production of reactive oxygen species (ROS), which directly damage mitochondrial DNA and proteins (Noren Hooten and Evans, 2021; Boyapati et al., 2017). Certain probiotics are able to reduce oxidative stress damage to mitochondria by increasing the levels of antioxidant molecules, such as glutathione (Xu et al., 2017). The gut microbiota produces inflammatory factors (e.g., tumor necrosis factor alpha (TNF-*α*), interleukin 6 (IL-6), etc.), and the upregulation of inflammatory factors can trigger mitochondrial damage in neurons (Feng et al., 2018; Sun et al., 2022). The stimulation of inflammatory factors also enhances neuronal mitochondrial oxidative stress (Semenova et al., 2024), generating excessive free radicals, which directly damage mitochondrial membranes, DNA and proteins and reduce mitochondrial function (Figure 3).

In summary, the gut microbiota regulates neuronal mitochondria through multiple pathways and signaling mechanisms. This regulation is inextricably linked to mechanisms such as immunoinflammatory, energy metabolism, mitochondrial autophagy, and oxidative stress.

### 4.3 Gut microbiota modulation of neuronal mitochondria affects depression

**Figure 4:**
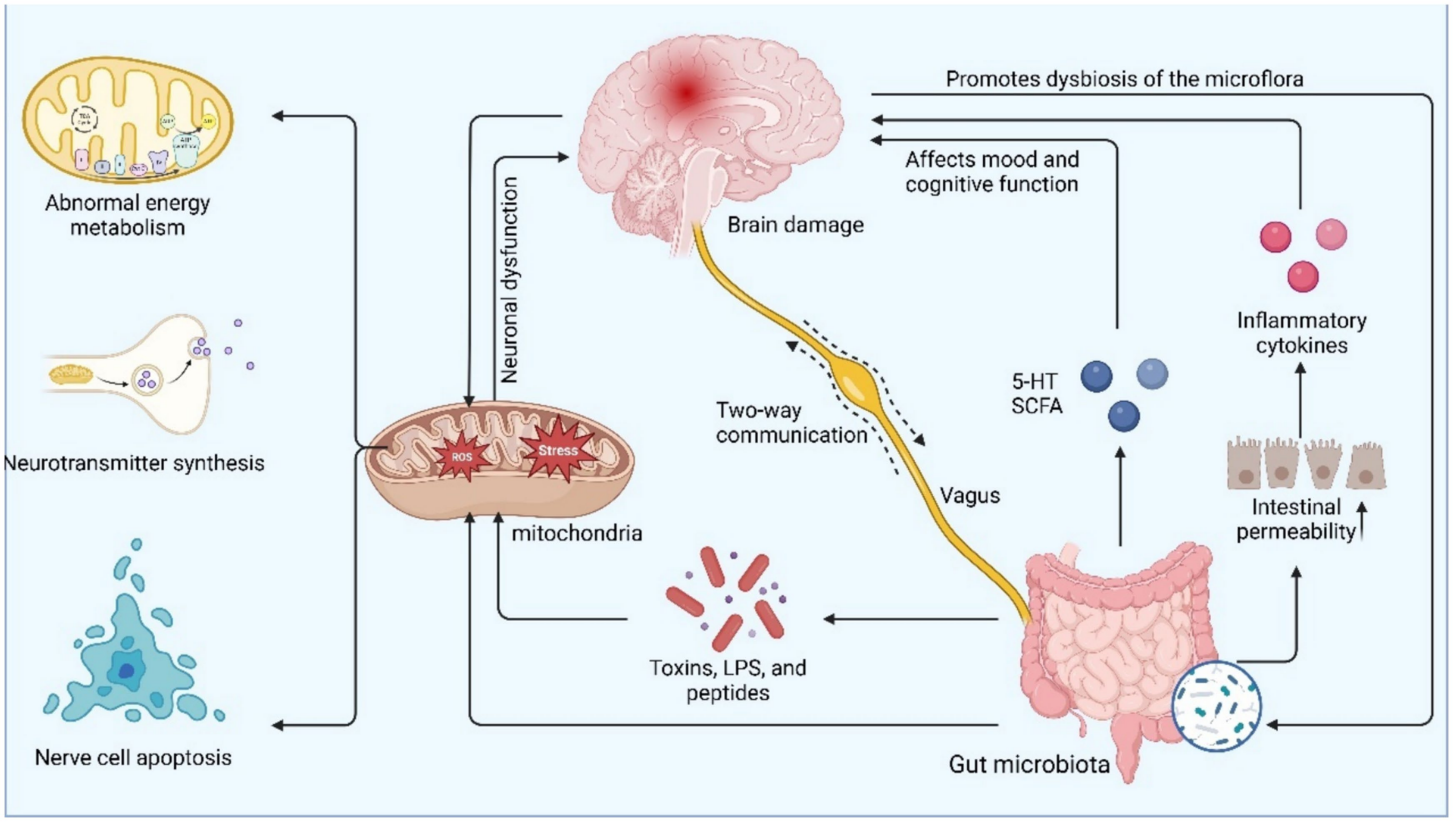
Gut microbiota modulates neuronal mitochondria, affecting depression.

The mechanisms underlying the correlations among mitochondria, the gut microbiota and depression are complex and multilayered. Mitochondria act as the energy factories of neurons and are responsible for ATP production. Their dysfunction may lead to energy deficits that affect neuronal health and are involved in the regulation of apoptosis and oxidative stress, processes that are closely associated with depression (Wu et al., 2021). The gut microbiota enter the brain through their metabolites (e.g., short-chain fatty acids, bile acids, amino acids, etc.) to affect neuronal and thus mitochondrial function (Cheng et al., 2024; Portincasa et al., 2022), and a deficiency of short-chain fatty acids may lead to impaired mitochondrial function, which in turn is able to affect metabolic and neurological function in the brain and exacerbate depressive symptoms. Imbalances in the gut microbiota (e.g., a decrease in probiotics and an increase in pathogenic bacteria) may lead to impaired mitochondrial function in neurons, which further affects energy metabolism in the nervous system, increases oxidative stress, and in turn promotes the development of depression (Irum et al., 2023). Depression is often accompanied by neuroinflammation, which causes nerve damage affecting mitochondrial function, and mitochondrial dysfunction exacerbates this inflammatory response, which in turn affects the functioning of brain regions (e.g., the hippocampus and prefrontal lobes), leading to the development of depression (Sittipo et al., 2022). Imbalances in the gut microbiota may increase intestinal permeability, contributing to the entry of endogenous inflammatory factors into the circulation, which further affects neuroinflammation in the brain (Sampson and Mazmanian, 2015). Oxidative stress is also an important factor, of which mitochondria are a major source, and excessive reactive oxygen species (ROS) generated by mitochondrial dysfunction within nerves can damage neurons and lead to neuronal dysfunction, whereas the gut microbiota can influence the level of oxidative stress by regulating the expression of antioxidant enzymes (Liu et al., 2023). The gut microbiota can influence neurotransmitter synthesis and metabolism through their metabolites, such as 5-HT (serotonin) synthesis, by regulating tryptophan metabolism. Mitochondria play important roles in the synthesis and metabolism of neurotransmitters, and mitochondrial dysfunction may further affect mood regulation by altering these metabolic pathways and has been associated with the development of depression (Cryan and Dinan, 2012; Bano et al., 2024). Intervention with the gut microbiota to modulate neuronal mitochondria is highly likely to be a target for certain brain-protective drugs (Stoccoro and Coppedè, 2021; Franco-Obregón and Gilbert, 2017) (Figure 4).

In summary, there is a close link between the gut microbiota and mitochondrial function, which regulates mitochondrial function and neuroinflammatory responses through metabolites, affecting energy metabolism and neurotransmitter synthesis in the brain, which in turn affects mood and behavior. Conversely, mitochondrial dysfunction can alter the composition of the gut microbiota. Therefore, the ability of the gut microbiota to regulate neuronal mitochondria through multiple pathways may provide new directions for the treatment of depression.

## 5 Summary

Depression, a major mental health disorder worldwide, places a heavy burden on patients and society. Current treatments face many challenges, including inadequate treatment rates, limitations of existing therapies, and high relapse rates. In recent years, the roles of the gut microbiota and mitochondrial dysfunction in the development of depression have gained increasing attention.

In this study, we systematically elucidated the molecular mechanisms by which the gut microbiota affects depression by regulating neuronal mitochondrial function through multiple pathways, revealing the important role of the gut microbe–mitochondrion–brain axis in the development of depression. The gut microbiota directly affects energy metabolism, the autophagy process, the oxidative stress balance, and the epigenetic modification of neuronal mitochondria through metabolites (e.g., short-chain fatty acids, neurotransmitters), immunomodulation, HPA axis regulation, and neurotransmitter metabolism. Among them, short-chain fatty acids such as butyric acid enhance mitochondrial biosynthesis through activation of the AMPK-PGC1α pathway while regulating mitochondrial autophagy to remove damaged organelles; colony-derived neurotransmitters such as GABA and 5-HT maintain neuronal function by regulating mitochondrial calcium homeostasis and oxidative phosphorylation efficiency; and endotoxemia and neuroinflammation triggered by imbalance of the gut microbiota are generated through ROS overproduction, mitochondrial DNA leakage and NLRP3 inflammasome activation exacerbating mitochondrial dysfunction. These findings provide a theoretical framework for the pathogenesis of depression from a “gut-brain-mitochondrial” perspective. Regulating the gut microbiota and thus neuronal mitochondrial function may provide a new strategy for the treatment of depression, and this mechanism of regulating neuronal mitochondria via multiple pathways provides new targets for the prevention and treatment of depression. Current therapeutic strategies for depression have expanded from traditional pharmacological interventions to multitargeted modulation of the gut microbiota–mitochondrial axis. In terms of treatment, emerging therapies such as phage therapy, engineered bacteria, and mitochondrion-targeted antioxidants (e.g., MitoQ) have also demonstrated great potential, in addition to probiotic, dietary, fecal transplantation, and mitochondrial transplantation approaches (Jiao et al., 2024; Mu et al., 2025). Phage therapy utilizes phages to lyse pathogenic bacteria and reduce infection density for the purpose of treating and preventing disease, which is especially important in dealing with antibiotic-resistant infections (Gordillo Altamirano and Barr, 2019). Engineered bacteria can be genetically engineered to perform special functions to overcome the shortcomings of traditional therapies and enhance therapeutic effects (Riglar and Silver, 2018). MitoQ, a mitochondrion-targeted antioxidant, can effectively reduce the amount of ROS produced by mitochondria and alleviate oxidative stress and has shown promising results in improving spatial memory function and mitochondrial respiratory function in disease models such as AD and ALS (Escribano-Lopez et al., 2019). However, the translation of mitochondrial transplantation and ketamine into clinical practice still faces several challenges; for example, the delivery method of mitochondrial transplantation needs to be optimized to ensure that exogenous mitochondria can effectively reach target tissues and perform their functions, and in terms of safety, the immune response and long-term effects triggered by mitochondrial transplantation need to be further investigated to ensure its safety and efficacy in clinical applications.

Although current studies have revealed the important role of the gut microbiota and mitochondrial dysfunction in depression, several limitations and knowledge gaps remain. Most of these studies are cross-sectional and do not reflect the dynamic changes in mitochondrial autophagy disorders, and the small number of clinical samples, mostly basic studies and different modalities of depression modeling, have led to inconsistent findings. In addition, few studies related to antidepressants and mitochondrial autophagy exist, and the specific molecular mechanisms of gut microbiota–mitochondrial interactions have not been fully clarified, especially in humans, where the long-term effects are unknown. In the future, longitudinal studies should be conducted to track the changes in the composition of the gut microbiota and mitochondrial function of patients with depression at different stages of the disease; individualized therapeutic targets should be clarified through clinical multi-omic cohort studies; multitargeted strategies integrating metabolic interventions and synergism between traditional Chinese and Western medicines should be developed; and the temporal and spatial dynamics of the bacterial–mitochondrial interaction should be analyzed with the help of organoid models, synthetic biology, and interdisciplinary technologies to break through the existing paradigm. This will break through the existing research paradigm and promote the transformation of depression treatment from “symptom control” to “mechanism repair” in precision medicine.
